# Vergesellschaftung unter Ansteckenden – für eine Körpergeschichte der Seuche

**DOI:** 10.1007/s00048-020-00253-9

**Published:** 2020-05-11

**Authors:** Fritz Dross

**Affiliations:** Institut für Geschichte und Ethik der Medizin, Friedrich-Alexander-Universität Erlangen-Nürnberg, Glückstr. 10, 91054 Erlangen, Deutschland

**Keywords:** Seuche, Kommunikation, Krankheit, Infektion, Epidemic, Communication, Disease, Infection

## Abstract

Dieser Beitrag ist Teil des Forums COVID-19: Perspektiven in den Geistes- und Sozialwissenschaften. Seuchen sind als „Menschheitstrauma“ seit eh und je Gegenstand der Geschichtswissenschaft für alle Epochen. Auf der Grundlage von Rudolf Schlögls Konzept der „Vergesellschaftung unter Anwesenden“ möchte ich im Folgenden vorschlagen, Seuchengeschichte als Körpergeschichte einer Kommunikation zu fassen, die das Kontagium als Gegenstand der Kommunikation einschließt.

## Abstract (en)

This paper is part of Forum COVID-19: Perspectives in the Humanities and Social Sciences. Being a “trauma of mankind” epidemics have been a major subject of historical research for a long time and regarding every historical period. Recurring to the concept of Rudolf Schlögl (“Vergesellschaftung unter Anwesenden”) my proposal is to research epidemics as a history of the communicating body and thus including the contagium as part of this communication.

Seuchen treffen den Menschen und das Menschliche dort, wo es am Empfindlichsten ist: „Seuchen sind die sozialsten aller Krankheiten.“ (Thießen 2015: 11) Und das in zweierlei Hinsicht: als übertragbare bzw. für übertragbar gehaltene Erkrankungen einerseits verbreiten sie sich im sozialen Kontakt, aus Verständigung wird Gefährdung durch Kommunikation. Andererseits zielen die angesichts von Seuchen ergriffenen Maßnahmen auf kollektives Verhalten, das Soziale mithin.

## Seuche – ein Kommunikationsphänomen

Die Differenzierung von Kommunikation zwischen Anwesenden und Abwesenden hat in diesen Ostertagen des Jahres 2020 eine sehr alltägliche Plausibilität gewonnen. Der weitgehende Verlust von Interaktionsformen, in denen sich „Alter und Ego […] als Körper in einer Welt wahr[nehmen], in der dieser Körper selbst und in seinem Bezug zu anderen Gegenständen und Körpern beobachtet werden kann“ (Schlögl 2008: 165), ist schmerzhaft. Wo der Mensch dem Menschen – *quom qualis sit non novit* –1 zum Wolf wird, stehen basale Formen der sozialen Interaktion auf dem Prüfstand. „Seuche“ – das ist in mehrfacher Hinsicht ein Phänomen der Kommunikation:

1. Es liegt auf der Hand, dass Seuchen solche Erkrankungsphänomene sind, die übertragbar sind, die kommuniziert werden. Krankheiten werden von Körpermedien kommuniziert, werden von den Kommunizierenden ausgetauscht.
2. „Seuche“, das ist gleichzeitig der Modus, in dem angesichts von massenhaftem Erkranken und der Furcht davor gesprochen, geschrieben und verordnet, komponiert und gesungen, gezeichnet, entworfen und gebaut wird.
3. Als Metakommunikation werden Vorstellungen und Theorien von der kommunizierbaren und kommunizierten Krankheitsmaterie entwickelt, modifiziert und verworfen.
4. Damit begründet wird die Reorganisation der Interaktion mit dem Ziel, das Kommunizieren der Erkrankung („die Ansteckung“) auszuschließen.
5. Der somit begründete Verzicht auf zentrale Sozialisationsinstanzen vom feierlichen Begräbnis bis zum samstäglichen Shopping, führt zu einer Krisenkommunikation.

Alfons Labisch beobachtete, dass öffentliche Wahrnehmung und Kommunikation kaum proportional zu Morbidität oder Letalität von „skandali(si)erten Krankheiten“ stehen. „Eine öffentliche Hysterie also – verästelt bis in die kleinsten Abläufe des Alltags! […] die Krankheiten indes, an denen die Menschen wirklich sterben, werden nicht erwähnt.“ (Labisch 2005: 274) Offenbar gibt es jeweils historisch ergänzende Faktoren, die aus einer Krankheit und deren Morbiditätsraten einen Skandal machen. Klar wird daraus auch: Die Seuchengeschichte befasst sich nicht mit Krankheiten (wie die Medizin), sondern mit Menschen, konkret: in den überlieferten Zeugnissen mit Wahrnehmungs- und Kommunikationsphänomenen des gefährdeten, beschädigten oder massenhaft beendeten Lebens. Sie analysiert Körperdiskurse über gleichzeitig gefährdete und gefährdende Körper.

Seuchendiskurse entstehen vermittels Kommunikation als „Hervorbringung von sozialem (das heißt, für die Beteiligten relevantem) Sinn unter Bedingungen doppelter Kontingenz“ (Schlögl 2008: 162; Schlögl 2014), und umfassen die kommunizierbare Seuche als unsichtbares Drittes. Vom Verbot feierlicher Hochzeiten und Begräbnisse bis zur Schließung eines Spielplatzes oder Flughafens steht bis dahin Unverhandelbares auf dem Prüfstand. Es geht um Aushandeln von Ordnung in der Krise, um Herrschaft als krisenhafte Praxis (Lüdtke 1991; Dinges 1995; Ulbricht 2004; Meier 2005).

## Sterben und Überleben

Am Anfang jeder Seuche steht das große Sterben. Dabei ist es unerheblich, ob sich *post factum* herausstellt, dass ganz andere Todesursachen in einem bestimmten Zeitabschnitt *de facto* häufiger waren. Zu unterscheiden sind langfristig hohe Sterberaten (etwa: Säuglingssterblichkeit) von katastrophisch auftauchendem und kommuniziertem Sterben. Die Seuchengeschichte ist bislang zu wenig als Teil der Geschichte der Katastrophen und ihrer Kommunikation betrachtet worden (Jankrift 2003). Würden wir uns ernsthafter mit der Wahrnehmung und Kommunikation der Zeitgenossen befassen, käme auch mittelalterliches Seuchen- als kommunikatives Geschehen massenhaft wieder ins Bild. Gängige Überblickswerke verbreiten das falsche Bild, zwischen der „Justinianischen Pest“ im 6. Jahrhundert, die auch als „Ende der Antike“ gedeutet wurde, und dem „schwarzen Tod“ in der Mitte des 14. Jahrhunderts, der seinerseits den Beginn eines Endes des Mittelalters markiert, hätten keine „Seuchen“ stattgefunden, weil zu selten von der uns vermeintlich bekannten „Pest“ die Rede ist (Jankrift 2005; Jankrift 2020).

Massenhaftes Sterben war in der langen Vormoderne ein in allen Generationen bekanntes Phänomen. Übliche Todesursache: Hunger. Sowohl mit Seuchen als auch mit Hunger stets in einem Zug ist vor allem für die Frühe Neuzeit der Krieg zu nennen – drei apokalyptische Reiter (Cunningham & Grell 2000). Tief in die kollektive Erinnerung eingedrungen ist der Zusammenhang insbesondere im Zusammenhang mit dem Dreißigjährigen Krieg (Bähr 2013).

Dass mangelernährte Menschen, untergebracht in erbärmlichen Behausungen, leicht Opfer von (Infektions‑) Krankheiten werden, sich unter diesen Bedingungen schwerere Verläufe einstellen und erheblich höhere Todesraten, war bereits im 16. Jahrhundert geläufig und bedarf keiner weiteren Hinweise auf „mangelnde Hygiene“. Bände spricht der Nürnberger Chronikeintrag für das Jahr 1544 über einen „kleinen Sterb“: „Sein aber nur viel arme Leut gestorben.“ (Müllner 2003: 642) Nördlinger Ärzte unterschieden 1571 zwei „genere pestis“, von denen eine lediglich die nach Hungerkrisen geschwächten Armen betrafen, während die andere gefährlich war, weil sie leicht auch die Wohlhabenderen „anstecken“ konnte (Kinzelbach 2006). Das Zählen macht den Unterschied als Wahrnehmungs- und kommunikative Praxis der Kontingenzbeherrschung.

Ein davon abweichendes Bild bietet die Influenza-Epidemie 1918–1920 in weiten Teilen Deutschlands (Rengeling 2017), die erst in der historischen Betrachtung zur „skandalisierten“ Seuche gemacht wurde. Das Geschehen lässt sich nur vor Ort studieren, und erneut wurde in Stadt und Land sehr unterschiedlich gezählt (Angerer et al. 2019; Witte 2018; Thießen 2016).

Die Medizingeschichte ist weitgehend davon abgekommen, historisches Massensterben auf dem Wege der „retrospektiven Diagnose“ unbekümmert modernen Krankheitsbegriffen zuzuordnen (Krischel 2019; Metzger 2014; Cohn 2013). Allerdings sind Vorgänge, für die sich keinerlei Analogien im zeitgenössischen terminologischen Apparat finden, größtenteils nicht untersucht, wie etwa der rätselhafte Nürnberger „portzel“, dessen Todesopfer für das Jahr 1483 mit über 4000 in der Chronistik vermerkt wurden (Dross 2016).

In der langen Geschichte der Vormoderne waren Menschen indes üblicherweise Überlebende von Seuchen – jede 40-jährige Person hatte wohl mindestens zwei schwere „Sterbsläufte“ überlebt. Die Geschichtswissenschaft hat sich dem Aspekt des Überlebens von Seuchen bislang nicht hinreichend gewidmet (Henderson 2019). Seuchen treffen uns im 21. Jahrhundert dagegen unvorbereitet, alle Strategien der (post-?) modernen „Risikogesellschaft“ versagen (Contzen et al. 2018).

## Gesund und krank

„Infektionstheorien“ unterlaufen herkömmliche Begriffe von „gesund“ und „krank“. Weder die betroffene Person noch ihr Gegenüber kann zuverlässig einschätzen, ob ihr oder der Körper ihres Gegenübers bereits „gefährlich“ ist. Infektionstheorien erzeugen also soziale Unsicherheit, um sie anschließend zu erklären. Das Sprechen darüber im Duktus des Krieges und die exzeptionelle Dramatik eines „unsichtbaren Feindes“ (Gradmann 2007; Berger Ziauddin 2009) kam mit der Bakteriologie zum Ende des 19. Jahrhunderts in die Medizin. Dort werden auch Visualisierungsstrategien deutlich, die dem unsichtbaren Feind einen Körper und ein Gesicht geben (Sarasin 2004).

Die Vormoderne verfügte über den Begriff eines „Miasma“ im Sinne einer Unreinheit, Befleckung oder auch Färbung, der fortwirkt. Aus dem individuellen Makel, der die Gemeinschaft, speziell die Gemeinschaft mit Gott schädigte, konnte abstrahierend eine ungünstige Eigenschaft der Luft gewonnen werden, die das gleichzeitige Erkranken mehrerer Personen innerhalb eines Bezirks gut erklärte (Potter 2005; Gudermann 2008). Ergänzend konnte ein durch Berührung oder die Luft vermitteltes „Kontagium“ im Sinne eines Ansteckungsgifts konkretisiert werden (Leven 2005). Insbesondere von Exkrementen und den Pestleichen, aber auch aus den Körpern der Erkrankten konnte erneut eine Luftverunreinigung an die Umgebung kommuniziert werden. Kultisch-rituell begründete Berührungsverbote sind von einer „medizinischen“ Ansteckungstheorie nicht trennscharf zu scheiden.

Unabhängig von der jeweils zu Grunde gelegten Übertragungstheorie figuriert der Ansteckungsstoff vom Krankheitssamen der Vormoderne bis zu Bakterium und Virus der Moderne über die Jahrhunderte als das im fremden Körper versteckte Andere. Besonders deutlich wird dies in imperialen Kontexten (Zeheter 2016; Kreuder-Sonnen 2015). Der erste Versuch jeder Seuchenpolizei gilt dem Verhindern des Zuzugs von außen. In der kommunikativen Praxis wird Identität weiterhin und bis heute als die Gemeinschaft der Reinen kommuniziert (Douglas [1966] 2001). Das schafft Identität bis in die Papierform – die seit dem 16. Jahrhundert ausgestellten, geprüften (und bald auch gefälschten) Zeugnisse, aus einer „unverdächtigen“ Gegend angereist zu kommen, können als maßgebliche Vorläufer moderner Identitätsausweise gelten (Groebner 2004).

Bemerkenswert ist die seit Jahrhunderten beobachtbare Fokussierung auf medizinische Spezialexpertise bei der Umgestaltung öffentlicher Ordnung. Dies ist umso bemerkenswerter, als die Medizin kurativ immer schon vergleichsweise hilflos war (wenn auch nie untätig!). Deutlich wird dies erst werden, wenn Normsetzung als soziale Praxis der Autorisierung einerseits analysiert wird, vor allem aber deren Praxis in Akzeptanz, Resilienz und Renitenz präziser gezeichnet sind (Ulbricht 2004: 36–59; Dinges 1995: 85–97).

Seuchenkommunikation erlaubt über die Jahrhunderte die Analyse eines Metaordnungsprinzips „Gesundheit“, das andere, sonst als zentral geltende Ordnungskriterien wie „Gerechtigkeit“ oder „Freiheit“ situativ dominiert, ohne in den herrschenden Ordnungsdiskursen, der jeweiligen Gesellschaftstheorie expliziert zu sein. Prävention als Verhaltenssteuerung und sozialräumliche Organisation indes beginnt mit der antiken Diätetik und war als obrigkeitliche Politik auch vor dem „schwarzen Tod“ bereits gängig (Geltner 2019).

Seuchenpolizei aber bedeutet noch immer auch deren Scheitern. Die Scheidung der „Reinen“ von den „Unreinen“ funktioniert nicht. In der Seuchenkommunikation werden aus „Gesunden“ „Noch-nicht-Erkrankte“, denen das Bestatten ihrer Angehörigen und der Besuch von Gottesdiensten wie von Volksfesten verboten wird, das Reisen erschwert oder untersagt, (nur) sie werden geimpft (Thießen 2017), sie werden zu „Gefährdeten“, „Verdächtigen“ und „Gefährdern“. So lässt sich nicht zusammen leben.

## Footnotes

1 „lupus est homo homini, non homo, quom qualis sit non novit.“ Plautus, Asinaria, 495. Ein Wolf ist der Mensch dem Menschen, kein Mensch, solange er nicht weiß, welcher Art der andere ist.
